# Incidence of hepatitis C among people who inject drugs in Ireland

**DOI:** 10.1186/s41124-017-0024-1

**Published:** 2017-01-26

**Authors:** Anne Marie Carew, Niamh Murphy, Jean Long, Kate Hunter, Suzi Lyons, Cathal Walsh, Lelia Thornton

**Affiliations:** 1 Health Research Board, Grattan House, 67-72 Lower Mount Street, Dublin, 2 Ireland; 2 HSE Health Protection Surveillance Centre, 25-27 Middle Gardiner Street, Dublin, 1 Ireland; 3 Department of Statistics, Trinity College Dublin, Dublin, Ireland; 4 Health Research Institute (HRI) and MACSI, University of Limerick, Limerick, Ireland

**Keywords:** Hepatitis C, Ireland, Injecting drug use, PWID, Incidence

## Abstract

**Background:**

Comprehensive information on the incidence and duration of hepatitis C virus (HCV) infection for people who inject drugs (PWID) in Ireland is not available. We created an incidence curve of injecting drug use in Ireland and subsequently estimated incidence of hepatitis C virus (HCV) infection.

**Methods:**

Anonymised data from the National Drug Treatment Reporting System (NDTRS) were used to identify all people who inject drugs (PWIDs) and who entered drug treatment for the first time between 1991 and 2014. A curve, estimating the incidence of injecting, was created to plot PWIDs by year of commencing injecting. The curve was adjusted for missing data on PWIDs in treatment and for PWIDs who were never treated. An adjustment was made to account for injectors who had never shared injecting equipment. The incidence of HCV infection and chronic infection in PWIDs was estimated by applying published rates.

**Results:**

Between 1991 and 2014, 14,320 injectors were registered on NDTRS. The majority were young (median age 25 years), male (74%), lived in Dublin (73%) and injected an opiate (e.g. heroin) (94%). The estimated total number of injectors up to the end of 2014 was 16,382. An estimated 12,423 (95% CI 10,799-13,161) were infected with HCV, and 9,317 (95% CI 8,022-9,996) became chronically infected. The estimated annual number of new HCV infections among PWIDs increased steeply from the late 1970s and peaked in 1998. By 2014, almost 30% of injectors were estimated to have been infected for over 20 years.

**Conclusions:**

This is the first comprehensive national estimate of the incidence of HCV in PWIDs in Ireland and will inform planning and developing appropriate health care services.

**Electronic supplementary material:**

The online version of this article (doi:10.1186/s41124-017-0024-1) contains supplementary material, which is available to authorized users.

## Background

Hepatitis C (HCV) was first identified in 1989. The acute phase of infection is usually asymptomatic, but approximately 75% of those who are infected develop chronic infection, which can cause liver cirrhosis, hepatocellular carcinoma (HCC) and liver failure [1, 2]. The risk of developing cirrhosis or HCC is higher in males, those who consume excess alcohol, those with hepatitis B or HIV co-infection [1] and those who were infected at older ages [2]. Between 5 and 20% of those who are chronically infected will develop cirrhosis after approximately 20 years of infection. Of those with cirrhosis, approximately 4% progress to decompensated liver disease each year and 1.6% develop HCC annually [2].

The main modes of transmission of HCV infection in Ireland are injecting drug use and the receipt of contaminated blood or blood products before the introduction of routine screening for HCV in the early 1990s. Information on the epidemiology of HCV in Ireland is mostly available from National Virus Reference Laboratory (NVRL) data since 1989, routine surveillance data based on statutory laboratory and clinical notifications to the Health Protection Surveillance Centre (HPSC) since 2004, and special studies in high prevalence groups such as people who inject drugs (PWIDs) and prisoners.

A study using NVRL diagnostic data and HPSC notification data estimated that approximately 10,000 individuals had been diagnosed with HCV and were living with chronic infection in Ireland by the end of 2009 [3]. The extent of underdiagnosis in Ireland is not known, but assuming levels of between 50 and 67% would increase this estimate to between 20,000 and 30,000 individuals [4]. Drug use was the most likely risk factor in 80% of the cases of HCV diagnosed by the NVRL between 1989 and 2004, of which 53% were genotype 1 and 42% genotype 3 [3]. A further 4,813 cases of HCV were notified to HPSC between 2010 and 2014 and, where risk factor data were available, approximately 80% were PWIDs [5].

The most recent capture-recapture study found that there were approximately 12,000 known opiate users (ever-injectors and non-injectors) in Ireland in 2006 and estimated the total number of known and ‘hidden’ opiate users to be 20,790 (95% CI 18,136-23,576) [6, 7]. Testing for blood-borne viral infections is offered to PWIDs in Ireland when they first attend opiate substitution services (clinics, prisons, and general practice) and is repeated at regular intervals. An Irish opiate treatment outcomes study commissioned in 2002 showed that HCV testing was carried out for 82% of attendees entering treatment services in rural, urban and inner-city areas [8]. However, addiction treatment services were not widely available outside of Dublin until more recent years and the proportion of PWIDs with undiagnosed HCV infection is likely to be higher outside of Dublin. Studies of PWIDs in prisons, opiate substitution services and general practice in Ireland, between 1992 and 2006, estimated the HCV antibody prevalence in this population to be between 52 and 84% [9–17]. Using a weighted average of the estimates from these studies (based on sample size), the national prevalence of HCV in PWIDs was calculated to be approximately 70% [16]. More recently, a cross-sectional study in 2011 reported lower levels of HCV antibody positivity in the prison population, with 41.5% of those with a self-reported history of ever injecting drugs testing positive for anti-HCV [18]. Of note, the prevalence of HCV infection differed depending on the type of drug injected; for example, 54% (80/149) of heroin injectors, 66% (66/100) of cocaine injectors and 62% (42/68) of benzodiazepine injectors tested HCV antibody positive, whereas only 21% (14/66) of steroid injectors tested positive [19].

The incidence and duration of HCV infection in Ireland have never been comprehensively described. The aim of this study was to estimate the incidence of HCV infection among PWIDs in Ireland. The objectives were: (1) to create an incidence curve of injecting drug use, (2) to estimate the annual incidence of HCV in PWIDs and (3) to estimate the number of PWIDs who developed chronic HCV infection.

## Methods

Anonymised individualised data from the National Drug Treatment Reporting System (NDTRS) formed the basis of this study. The NDTRS records information on treated problem drug use in Ireland using an agreed Europe-wide protocol (http://www.emcdda.europa.eu/publications/manuals/tdi-protocol-3.0) and covers 76% of drug and alcohol treatment services in Ireland. It was established in the Greater Dublin Area in 1990 by the Health Research Board and was extended in 1995 to cover all areas of the country [20]. Treatment data for problem drug use in Ireland are provided to the NDTRS by statutory and non-statutory services, including outpatient services, residential centres and general practices. Clients who attend needle-exchange services are not included in this reporting system. The database records client information on the date of treatment, whether a client has ever previously received drug treatment (incident case identifier), ever injected drugs, age when they first injected, and recent injection behaviour, in addition to detailed demographic, drug use and treatment data. The NDTRS does not contain information on HCV status.

Injecting and HCV infection curves were created using a four-stage process.

1. **Original injector data and imputed missing values**
  In 2016, all ever-injector individuals, whether current or former injectors, entering drug treatment for the first time between 1991 and 2014 were identified and extracted from the NDTRS. The length of time an individual injected before coming to their first treatment as an injector was ascertained and an injecting curve was created for the number of cases starting to inject each year. The year of commencing injecting was missing for a proportion of cases. Factors associated with missing values were investigated and discovered not to be completely random. Therefore, in order to account for this, missing data were generated using Expectation-Maximization (EM) methods. The original and imputed data were combined to create an epidemic curve based on the year of commencing injecting for all PWIDs registered on the NDTRS up to the end of 2014.
2. **Estimating injectors not in treatment**
  Treated PWIDs represent a proportion of the injecting drug population. Estimates for the proportion of PWIDs not in treatment were used to adjust the epidemic curve for all PWIDs. In the absence of Irish data for this proportion, Scottish rates were applied to each yearly estimate of injectors residing in Dublin (6% of injectors never attended treatment in Glasgow between 2008 and 2010) [21]). Scottish data were considered the most appropriate as the drug situation in Dublin has been likened to that in Glasgow [22]. For injectors living outside Dublin, a worst case scenario estimate of 50% non-attendance was used based on the 2006 capture-recapture estimate [7], as appropriate harm reduction and treatment services were not historically available to a large proportion of these. No adjustment was made to take account of PWIDs who died prior to the commencement of the NDTRS in 1991. Using a Mann–Whitney *U* test, the estimated number of injectors was validated by comparing it with published data available for four time points, from capture recapture studies estimating the number of opiate users in Ireland [6, 7]. The number of opiate users who were injectors was derived using the known percentage of opiate injectors in the NDTRS.
3. **Estimating HCV infections**
  In order to adjust for injectors who were not at risk of infection a conservative 21% deduction was made from the total estimate of injectors to allow for those who had never shared injecting equipment. The 21% was based on the lowest annual proportion of cases (worst case scenario) who reported through the NDTRS that they had never shared and had been in treatment for drug use two or more times during the 23 year time period [unpublished NDTRS data]. In order to estimate the incidence of HCV infection among PWIDs, published HCV incidence rates in PWID populations for different time periods in Ireland were applied to the injecting curve. Incidence data were available for two time periods. Smyth et al. found a HCV incidence rate of 66 per 100 person years (95% CI 51–84) in a study carried out between 1992 and 1998 [23]. This rate and its 95% confidence intervals (CI) were used for those who commenced injecting between 1992 and 1998. In a study carried out in 2001, Grogan et al. [13] estimated that the infection rate for clients in opiate substitution services in the parts of Dublin and surrounding areas was 24 per 100 person years (95% CI 12.2–43.8) but included both injectors and non-injectors in their denominator. We adjusted this rate and its 95% confidence intervals using the proportion of opiate users entering treatment in the area in 2001 who were recorded in the NDTRS as having reported injecting (70%) and thus estimated the infection rate in PWIDs to be 35 per 100 person years (95% CI 17.4–62.6). This rate and its 95% confidence intervals were applied to 2001 and continued for the period 2001 to 2014 as these were the most recent estimates. As no published data were available for the period 1953 to 1991, the mid-point between the highest and lowest incidence and its confidence intervals for the two periods with known incidence rates were applied; these were a rate of 50.7 per 100 person years and 95% CI of 17.4–84. For 1999 and 2000 the mid-points of the rate and the upper and lower confidence intervals for the two periods with known incidence rates on either side of the timeline were applied (50 per 100 person years, and 95% CI 34.6–73.4).
4. **Estimating chronic HCV**
  In order to obtain an estimate of chronic HCV infections, an internationally accepted 75% conversion from acute to chronic HCV was applied to the HCV infections curve [2].

## Results

### Original injector data and imputed missing values

**Fig. 1:**
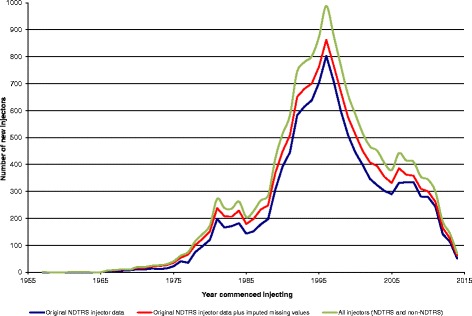
New injectors by year of commencing injecting

During the 23-year study period (1991–2014), 14,320 ever-injectors entered addiction treatment and were registered on the NDTRS. The majority were male (74.3%) and lived in Dublin (73.0%). The median age for injectors entering treatment was 25 years (90% central range 18–40 years) while the median age at commencing injecting was 20 years (90% central range 15–31 years). Half of all PWIDs had injected for 3 years or more before entering addiction treatment (90% central range 0–18 years). Opiates were the main problem drug reported by 94.1% of PWIDs. Seventy one per cent reported problem use of more than one drug and 14.7% had been in treatment as a smoker and later returned to treatment as an injector. Problem alcohol use was reported by 9.1% of injectors on entering treatment. Data necessary to calculate the year of first injecting were available for 86.4% (*n* = 12,375) and missing for 13.6% (*n* = 1,945). The most common missing information was age first injected (*n* = 1,929, 13.5%). Using logistic regression, year of treatment and main problem drug were found to be significantly associated with the probability of a patient’s record not having data on age first injected. An imputed year of first injecting was assigned to individuals with missing data using the pattern of observed data for similar injectors (Fig. 1).

The resulting injecting curve shows few new PWIDs in Ireland prior to 1970 (*n* = 36, 1957 to 1969); the estimated number of new PWIDs increased steadily in the late 1970s and then continued to increase at a lower rate into the 1980s (Fig. 1). Incidence of injecting increased again in the late 1980s and early 1990s, peaking in 1998. Data from 2011 to 2014 are likely to be incomplete due to the lag time between injecting and first attending treatment.

### Estimating injectors not in treatment

Adjusting for PWIDs who had never attended drug treatment services and therefore who were not registered on the NDTRS, added a further 2,062 injectors to the curve. The total estimated number of PWIDs up to the end of 2014 is 16,382 (Fig. 1).

Estimates from the injecting curve were validated against the number of opiate injectors estimated using opiate user data from Irish capture recapture studies over four different years [6, 7] and data on the percentage of opiate users who were injecting in those years from the NDTRS (Table 1). Overall the estimates were not statistically significantly different (U = 5.00, *p* = 0.49).

**Table 1:** Comparison of estimates from the injecting curve with published data from a capture recapture study

Year	Opiate users in capture recapture^a^	95% CI	% opiate injectors in NDTRS	Estimated opiate injectors in capture recapture	Opiate injectors calculated from injecting curve	% difference between capture recapture and injecting curve
1996	13461	12037–15306	65%	8750	8469	−3.2
2000	14158	12884-15883	83%	11751	11362	−3.3
2001	14452	13405–15819	78%	11273	11879	+5.4
2006	20790	18136–23576	65%	13513	14024	+3.8

^a^[6, 7]

### Estimated HCV infections

**Fig. 2:**
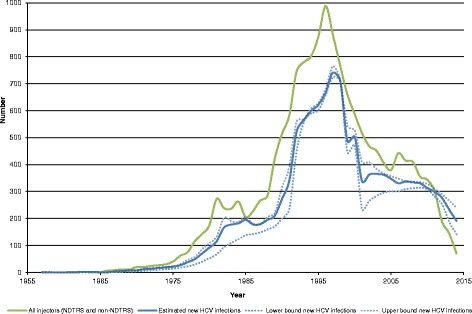
Estimates of new injectors by year commenced injecting and new HCV infections by year infected

**Fig. 3:**
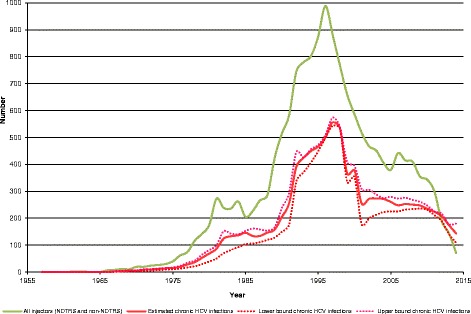
Estimates of new injectors by year commenced and chronic HCV infections by year infected

After adjusting for injectors who had never shared injecting equipment, it is estimated that 12,423 (95% CI 10,799-13,161) PWIDs were infected with HCV over the entire period (Fig. 2) and that 9,317 (95% CI 8,022-9,996) became chronically infected (Fig. 3). The estimated number of new infections peaked in 1997. By 2014, more than one quarter (27.0%) of PWIDs with chronic HCV infection were estimated to have been infected for 0–10 years, 43.4% for 11–20 years, 22.8% for 21–30 years and 6.7% for over 30 years.

## Discussion

This study is the first comprehensive national estimate of the incidence of injecting drug use in Ireland along with the incidence and extent of the hepatitis C epidemic in this population. Results show an estimated 16,400 PWIDs in Ireland during the 24 year study period (1991–2014). The majority of injectors were young, male, lived in Dublin and injected heroin. Over 12,000 PWIDs were estimated to have been infected with HCV by 2014, with over 9,000 likely to have become chronically infected. More than 40% of injectors were estimated to have been infected for 11–20 years by 2014 and almost 30% were estimated to have been infected for over 20 years. However, some of these injectors will have since died or may have been successfully treated for HCV infection and the cumulative incidence numbers presented in this paper do not take account of these events. In relation to deaths, the National Drug-Related Death Index reported that 937 PWID who were hepatitis C-infected died between the years 1998 and 2014 (Personal communication: Dr Suzi Lyons, Senior Researcher, National Health Information Systems, Health Research Board. 8 November 2016). In relation to treatment for hepatitis C, a national programme set up at the end of 2014 offering direct acting antivirals to individuals testing positive for hepatitis C and meeting pre-defined clinical criteria reported that 138 PWID were successfully treated (measured by a sustained virological response) between December 2014 and August 2016 (Personal communication: Michele Tait, Programme Manager, HSE Hepatitis C Treatment Programme. 3 November 2016). Prior to this, research published in 2007 indicated that few PWID were suitable for (13%) or commenced (3%) antiviral treatment [15]. Thus the overall numbers successfully treated or who died are likely to be small and do not change the overall estimate of the extent of the hepatitis C epidemic.

The overall size and pattern of the estimated hepatitis C incidence curve is similar to a hepatitis C infection curve based on the date of laboratory diagnoses, despite a different study approach [3]. Both studies found that hepatitis C incidence is now decreasing in Ireland. This is supported by routine surveillance data which show that statutory notifications have declined significantly since 2012 [5]. Given this fall in the number of newly diagnosed cases and the fact that the most recent incidence study dates to 2001, the authors acknowledge that there may be a corresponding overestimate in later years. As PWIDs make up the majority of known hepatitis C infected people in Ireland, the results from this study are of benefit in estimating the extent of the overall HCV epidemic in Ireland. The other known large cohort is people infected through blood and blood products in the past in Ireland. This is a historical cohort of over 1,700 people, approximately 390 of whom remained alive and chronically infected at the end of 2013 [24]. Undiagnosed infection in this population would be expected to be rare due to extensive national screening programmes. There may, however, be a significant number of undiagnosed cases of HCV in the recent migrant population in Ireland. Although asylum seekers are routinely offered infectious disease screening, there is no systematic testing for other migrants. At the time of the 2011 census, there were 766,770 non-Irish nationals living in Ireland. Based on census data on the number of people living in Ireland by country of birth (CSO unpublished data, Olive Pluck, CSO) and published data on the prevalence of anti-HCV by country of birth [25, 26], over 10,000 of these are likely to be chronically infected with HCV. This assumes that the prevalence of HCV in the migrant population in Ireland is similar to published data for the general population in their country of birth.

There are a number of limitations to the methods used in this study. The number of PWIDs who never accessed drug treatment was unknown and was estimated. However, the estimates of PWIDs were validated against capture recapture studies [6, 7] and were not statistically different. The hepatitis C infection rates that were applied were based on incidence studies during two specific time periods and varied considerably from 66 to 35%. This is not surprising as HCV incidence rates for Glasgow show similar variations although their injecting curve is approximately 10 years ahead of the Irish curve; for example, model-based rates estimated for Glasgow were 6–40, 78–89 and 18–30 per 100 injector-years during the periods 1960–1976, 1977–1986 and 1990–2000 respectively [27]. The decrease in the incidence of HCV infection in opiate users in Ireland may be due to the provision of opiate detoxification and substitution in prisons and the increased provision of such programmes in the community, in particular outside Dublin [28]. Another limitation was that we did not have data on whether individuals ceased to inject. Therefore HCV infection rates were applied to each individual each year. However, a proportion of individuals may have ceased injecting prior to becoming HCV infected and no longer have been at risk of infection. A proportion of individuals may also have died, and this is not reflected in the estimates described here. Our interval estimates in Figs. 2 and 3 account for known uncertainties in the inputs, but do not account for other uncertainties and modelling assumptions the impact of which is not explicitly known. Despite these limitations, as already stated, modelled curves are broadly in line with those described by laboratory diagnoses and by surveillance data [3, 5].

Modelling the progression of disease for the population is of interest from a public health perspective, especially in the context of emerging treatments for HCV. This work is ongoing by one of the authors (CW), involving the calibration of disease models, and evidence syntheses for treatments that are available, and is due for publication in the near future. The additive effect of alcohol use on progression of HCV-related cirrhosis [1] will be an important factor to take into account, as PWIDs in Ireland have been shown to have a high prevalence of problem alcohol consumption. Only 9% reported problem alcohol use on first entry to treatment; however, data on previously treated opiate users indicate that 21% reported problem alcohol use [18] and 35% of opiate users receiving methadone in general practice in Dublin were harmful alcohol users [29]. Recent drug treatment data show that the number of injectors entering drug treatment for the first time in Ireland has dropped slightly in recent years [30]. However, injecting drug use remains a significant issue. The European Monitoring Centre for Drugs and Drug Addiction (EMCDDA) recommends the collection of accurate data on the incidence of injectors entering treatment as this is an indicator of problem drug use [31]. Therefore, this analysis demonstrates the wider usefulness of routine drug treatment data collected by the NDTRS. It is important that harm reduction strategies continue to be implemented and evaluated in order to achieve reductions in injecting risk behaviours and HCV transmission. Although the incidence of injecting and the incidence of HCV have declined in recent years, unless substantial numbers receive treatment for their HCV infection the burden of HCV-related liver complications will be high over the next few decades. The information from this study will be of benefit in planning cost-effective approaches to the use of the highly effective but expensive direct acting antiviral treatments now available.

## Conclusions

This paper is the first comprehensive national estimate of the incidence of injecting drug use in Ireland, along with the incidence and extent of the hepatitis C epidemic in this population. The findings will inform those responsible for planning and developing health care services and will be of benefit in planning cost-effective approaches to the use of the highly effective but expensive new treatments for hepatitis C that have recently become available.

## Additional file

Additional file 1: Data and computations. (XLSX 15 kb)

## Abbreviations

EMCDDA: European Monitoring Centre for Drugs and Drug Addiction
HCC: Hepatocellular carcinoma
HCV: Hepatitis C virus
HPSC: Health Protection Surveillance Centre
NDTRS: National Drug Treatment Reporting System
NVRL: National Virus Reference Laboratory
PWID: People who inject drugs
